# Comparison of Genetic Variants in Cancer-Related Genes between Chinese Hui and Han Populations

**DOI:** 10.1371/journal.pone.0145170

**Published:** 2015-12-18

**Authors:** Chaoyong Tian, Zhiqiang Chen, Xixian Ma, Ming Yang, Zhizhong Wang, Ying Dong, Ting Yang, Wenjun Yang

**Affiliations:** 1 Cancer Research Institute of the General Hospital, Ningxia Medical University, Yinchuan, Ningxia, China; 2 Key Laboratory of Ningxia Reproduction and Heredity, Ningxia Medical University, Yinchuan, Ningxia, China; 3 Radiology Department of General Hospital, Ningxia Medical University, Yinchuan, Ningxia, China; 4 Chinese Academy of Sciences (CAS) Key Laboratory of Computational Biology, Max Planck Independent Research Group on Population Genomics, CAS-MPG Partner Institute for Computational Biology (PICB), Shanghai Institutes for Biological Sciences, Chinese Academy of Sciences, Shanghai, China; 5 Beijing Laboratory of Biomedical Materials, College of Life Science and Technology, Beijing University of Chemical Technology, Beijing, China; 6 Department of Epidemiology, School of Public Health, Ningxia Medical University, Yinchuan, Ningxia, China; Medical College of Soochow University, CHINA

## Abstract

**Background:**

The Chinese Hui population, as the second largest minority ethnic group in China, may have a different genetic background from Han people because of its unique demographic history. In this study, we aimed to identify genetic differences between Han and Hui Chinese from the Ningxia region of China by comparing eighteen single nucleotide polymorphisms in cancer-related genes.

**Methods:**

DNA samples were collected from 99 Hui and 145 Han people from the Ningxia Hui Autonomous Region in China, and SNPs were detected using an improved multiplex ligase detection reaction method. Genotyping data from six 1000 Genomes Project population samples (99 Utah residents with northern and western European ancestry (CEU), 107 Toscani in Italy (TSI), 108 Yoruba in Ibadan (YRI), 61 of African ancestry in the southwestern US (ASW), 103 Han Chinese in Beijing (CHB), and 104 Japanese in Tokyo (JPT)) were also included in this study. Differences in the distribution of alleles among the populations were assessed using χ^2^ tests, and F_ST_ was used to measure the degree of population differentiation.

**Results:**

We found that the genetic diversity of many SNPs in cancer-related genes in the Hui Chinese in Ningxia was different from that in the Han Chinese in Ningxia. For example, the allele frequencies of four SNPs (rs13361707, rs2274223, rs465498, and rs753955) showed different genetic distributions (p<0.05) between Chinese Ningxia Han and Chinese Ningxia Hui. Five SNPs (rs730506, rs13361707, rs2274223, rs465498 and rs753955) had different F_ST_ values (F_ST_ *>*0.000) between the Hui and Han populations.

**Conclusions:**

These results suggest that some SNPs associated with cancer-related genes vary among different Chinese ethnic groups. We suggest that population differences should be carefully considered in evaluating cancer risk and prognosis as well as the efficacy of cancer therapy.

## Introduction

Genetic studies have revealed that different populations have different genetic structures because of their complex demographic histories [1]. Therefore, genetic differences in cancer-related genes are expected to exist between different ethnic groups. This diversity in cancer-related genes may result in differences in cancer susceptibility, sensitivity to radiotherapy and chemotherapy as well as prognosis among different ethnic populations. For example, p53 is well known as the most commonly mutated gene in human cancer. Codon 72 of p53, localized in exon 4, is among the most intensively studied polymorphisms found in the coding region of TP53. Substitution of Arg (codon CGC) with Pro (codon CCC) at residue 72 (R72P) results in a structural change of the protein [2]. Banks et al. demonstrated the existence of biochemical and biological differences between the Arg and Pro isoforms of p53 [3]. Several groups have reported an association between the Arg p53 variant and increased risk of epithelial cancer such as gastric cancer [4]. However, other studies have shown the opposite correlation, with the Pro (lesser apoptotic) variant corresponding to an increased risk of other cancer types such as thyroid cancer [5]. Beckman et al. first noted a significant difference in the frequency of the Pro allele between a Nigerian population (African Black) and a Swedish population (Western European), with values of 17% and 63%, respectively [3]. The frequency of p53 codon 72 alleles and haplotypes differs across ethnicities, which may be the leading cause of the different effects of the p53 codon 72 polymorphism on cancer risk in different ethnicities [6].

There are 56 ethnic groups in China. Han is the largest ethnic population, comprising 98% of the total population in China. The populations of the other 55 minority groups vary from thousands to millions, and some of the minority groups differ substantially from Han Chinese in terms of morphological and genetic characteristics. For example, the Uyghur (UIG) population, the fifth largest minority group in China, differs significantly from the Han Chinese in terms of facial features and has an approximately 55% European genetic component [7]. Hui Chinese, behind only Mongolian Chinese, is the second largest ethnic minority with a population of more than 12 million. The majority of the Chinese Hui people live in Ningxia Hui Autonomous region (hereafter Ningxia) located in northwest China, accounting for one-third of the population in Ningxia [8]. It has been proposed that the Hui Chinese may have descended from Central Asian, Arab, and Persian merchants who came to China during the 7th century. Therefore, populations may differ from Han Chinese with respect to their genetic background. Single nucleotide polymorphisms (SNPs) have emerged as genetic markers of choice because of their high density and relatively even distribution across the human genome, and SNPs have been used for fine mapping of disease loci and for candidate gene association studies [9]. Several investigations have recently shown that differences existed in psychological stress susceptibility, cancer susceptibility and cancer prognosis between Chinese Hui and Chinese Han [10,11,12]. However, there are still a lot of works needed to be done to ultimately unveil the genetic differences between these two groups of people. In this study, we randomly selected eighteen SNPs in cancer-related genes to advance the understanding of the genetic differences between Hui and Han Chinese from the Ningxia region of China.

## Subjects and Methods

### 2.1 Genetic Variation Data

All subjects were from the Ningxia Hui Autonomous region in China. A total of 99 Hui individuals were selected from the physical examination center of a county hospital located in the Ningxia Haiyuan region of China. A total of 145 Han individuals were selected from the physical examination center of the General Hospital of Ningxia Medical University. The inclusion criteria were: (1) All subjects were from Ningxia Han or Hui residents whose ancestral native living places were Ningxia Hui Autonomous Region and at least three generations of their families were also the same ethnic people. (2) All subjects were proved to be physically healthy by their history, physical examination, and clinical examination when their samples were collected. (3) All subjects were proved to be free of benign or malignant tumors, both previously and at present, by their history, physical examination, and clinical examination. Demographic data including age, gender, and alcohol and tobacco consumption were obtained using a survey. Blood samples were collected from all subjects for DNA isolation and genotyping of the eighteen SNPs. Signed informed conset was obtained from each participant. All procedures were approved by the Medical Ethics Review Committee of Ningxia Medical University (Ningxia Region, China).

### 2.2 Cancer-Related Genes and Selected SNPs

Eighteen SNPs were selected for the analysis. Among all the SNPs, ten including rs13042395, rs465498, rs753955, rs17728461, rs2274223, rs13361707, rs9841504, rs9485372, rs4488809, and rs9934948 were reported by GWAS to be associated with cancer risk. For example, four SNPs associated with lung cancer are located in the *TP63* gene (rs4488809 at 3q28), *TERT*-*CLPTM1 L* gene (rs465498 at 5p15), *MIPEP*-*TNFRSF19* gene (rs753955 at 13q12), and *MTMR3*-*HORMAD2*-*LIF* gene (rs17728461 at 22q12). Two SNPs related to esophageal cancer are located in the *PLCE1* gene (rs2274223 at 10q23) and *C20orf54* gene (rs13042395 at 20p13). Two SNPs related to breast cancer are located in the *TAB2* gene (rs9485372 at 6q25) and *LOC100506172* (rs9934948 at chromosome 16). Two SNPs with independent effects and significant gastric cancer associations are located in the *PRKAA1* (rs13361707 at 5p13) and *ZBTB20* genes (rs9841504 at 3q13).

Eight other SNPs, including rs1042522, rs3176320, rs3829964, rs762624, rs4135234, rs730506, rs3829963, and rs2395655, are in p53 or *CDKN1A*, both of which play a critical role in carcinogenesis in the p53 pathway. For example, only one SNP, rs1042522, is located in the p53 gene (at 17p13), which represents one of the most studied tumor suppressor genes in cancer biology. A great number of genetic association studies have reported that rs1042522 is a risk factor for human malignancies [3,13]. Seven of the remaining SNPs are located in the promoter region of *CDKN1A* gene (rs2395655, rs3176320, rs3829964, rs762624, rs4135234, rs730506, and rs3829963 at 6p21), six of which have been analyzed for their associations with longevity, esophageal squamous cell carcinoma, or lung cancer except rs3176320 [14,15,16,17].

### 2.3 DNA Extraction and Genotyping

Genomic DNA was extracted using Qiagen genomic DNA extraction kits (QIAGEN Inc., Valencia, CA, USA). SNP genotyping was performed using an improved multiplex ligase detection reaction method (iMLDR, Genesky Bio-Tech Cod., Ltd., Shanghai, China) as previously described [18]. The primers and probes of ten SNPs used in polymerase chain reactions (PCRs) and ligase detection reaction (LDR) were listed in S1 Table, and the primers and the probes for the remaining eight SNPs used in PCR and LDR were the same as previously described [19].

Genotyping data from six 1000 Genomes Project population samples (99 Utah residents with northern and western European ancestry (CEU), 107 Toscani in Italy (TSI), 108 Yoruba in Ibadan (YRI), 61 persons of African ancestry in the southwestern United States (ASW), 103 Han Chinese in Beijing (CHB), and 104 Japanese in Tokyo (JPT)) were included in this study. According to two references by Xu et al. and Hu et al, we downloaded the genotype data of individuals from six populations from the 1000 Genomes Project web site (www.1000genomes.org) as controls [20,21]. These individuals derive from three different population groups covering six subpopulations: CEU and TSI as European groups, YRI as representation of Africans, ASW as African American, and CHB and JPT as East Asian groups.

### 2.4 Statistical and Population Genetic Analyses

Genotype and allele frequencies were obtained by direct counting. Differences in the distribution of alleles among the populations were assessed using the χ^2^ test. All significance tests were two-tailed and were considered statistically significant at p<0.05. Statistical Package for Social Sciences (SPSS) version 17.0 statistical software was used for the statistical analyses. The F_ST_ value, originally defined by Wright, was introduced as the correlation between gametes chosen randomly from within the same subpopulation relative to the entire population. F_ST_ can be thought of either as the proportion of genetic diversity due to allele frequency differences among populations or as the correlations between alleles within populations relative to the entire population [22,23,24]. F_ST_ calculations were performed using Arlequin 3.5. The F_ST_ of SNPs were calculated following Weir and Cockerham [7]. The F_ST_ of an SNP was two-tailed and were considered statistically significant at p<0.05.

## Results

We investigated a total of 244 subjects, including 99 Hui and 145 Han subjects. Table 1 shows the demographic characteristics, including age, gender, cigarettes smoking, and alcohol drinking. There were no significant differences in age, gender, and drinking consumptions. Compared with Hui people, however, there were more cigarette smokers in Han population than that in Hui population (32.4% vs 16.2%).

**Table 1:** Distributions of Selected Characterstics Between Chinese Ningxia Han and Hui.

		Ningxia Han(n = 145)	Ningxia Hui (n = 99)		
Variable		n %	n %	*χ* ^*2*^ */t*	*p value*
Gender	Male	85(58.6)	55(55.6)	0.226	0.634
	Female	60(41.4)	44(44.4)		
Age(mean±SD), years		44.96±12.852	43.56±10.621	-0.930	0.354
Cigarettes Smoking	Yes	47(32.4)	16(16.2)	8.113	0.004
	No	98(67.6)	83(83.8)		
Alcohol Drinking	Yes	23(15.9)	11(11.1)	1.107	0.293
	No	122(84.1)	88(88.9)		

To identify cancer-related genes that are highly differentiated with respect to allele frequency among the six 1000 Genomes Project populations and the two populations from the Ningxia region of China, an F_ST_ value, a measure of genetic differentiation, was calculated for each SNP to quantify the differences among the different populations. As shown in Table 2, all of the SNPs had different F_ST_ values among the eight populations, varying from 0.013 to 0.192, and all *p* values were less than 0.05. This finding suggests that the cancer-related genes differ substantially among the studied populations.

**Table 2:** SNPs that Are Differentiated among CEU, TSI, YRI, ASW, JPT, CHB, Chinese Ningxia Hui, and Chinese Ningxia Han.

rsID	Fst	p Value	Chr.	Position	Gene	MIM Number	Catagory
rs1042522	0.084	0.000	17	7579472	TP53	191170	exon4
rs2395655	0.087	0.000	6	36645696	CDKN1A	116899	5'-flanking
rs3176320	0.058	0.000	6	36646788	CDKN1A	116899	intron1
rs3829963	0.071	0.000	6	36644386	CDKN1A	116899	5'-flanking
rs3829964	0.103	0.000	6	36644498	CDKN1A	116899	5'-flanking
rs4135234	0.013	0.003	6	36644221	CDKN1A	116899	5'-flanking
rs762624	0.094	0.000	6	36645588	CDKN1A	116899	5'-flanking
rs730506	0.035	0.000	6	36645968	CDKN1A	116899	5'-flanking
rs4488809	0.024	0.000	3	189356261	TP63	603273	N/A
rs13042395	0.113	0.000	20	754511	C20orf54	613350	N/A
rs13361707	0.057	0.000	5	40791884	PRKAA1	602739	N/A
rs17728461	0.035	0.000	22	30598552	LIF downstream 38kb	N/A	N/A
rs2274223	0.049	0.000	10	96066341	PLCE1	608414	N/A
rs465498	0.192	0.000	5	1325803	CLPTM1L	612585	N/A
rs753955	0.171	0.000	13	24293859	MIPEP-TNFRSF19	N/A	N/A
rs9485372	0.082	0.000	6	149608874	TAB2	605101	N/A
rs9841504	0.062	0.000	3	114362764	ZBTB20	606025	N/A
rs9934948	0.134	0.000	16	73439355	LOC100506172	N/A	N/A

Abbreviations are as follows: CEU, Utah residents with Northern and Western European ancestry; TSI, Toscani in Italy; YRI, Yoruba in Ibadan; ASW, African Ancestry in Southwest US; JPT, Japanese in Tokyo; CHB, Han Chinese in Beijing; Chr., chromosome; MIM, Mendelian Inheritance in Man; N/A, not available.

The average F_ST_ value between each pair of subpopulations for the eighteen SNP sites was also calculated (Table 3). The F_ST_ values between CEU and TSI, YRI and ASW, and JPT and CHB varied from 0.00440 to 0.00970, showing that there was little genetic differentiation between any two European groups, two African groups, or two East Asian groups. The average F_ST_ value between CHB and Chinese Ningxia Han was 0.0000, which was less than the value between Ningxia Hui and Ningxia Han (0.00363). However, the average F_ST_ value between two different ethnic groups of people among the European groups, African groups, and two East Asian groups varied from 0.07961 to 0.16061, suggesting that there was considerable genetic differentiation. For example, the maximum average F_ST_ value was 0.16061 between CEU and YRI, and the minimum value was 0.07961 between ASW and JPT. We found that the average F_ST_ value between Chinese Ningxia Han and CEU showed the greatest differentiation (0.10627) between Chinese Ningxia Han and the other populations; furthermore, the average F_ST_ value between Chinese Ningxia Han and CHB showed the least differentiation (0.00000), and the value between Chinese Ningxia Han and Hui was between the maximum and minimum (0.00363). Similarly, regarding the differentiation between Chinese Ningxia Hui and the other populations, the average F_ST_ value between Chinese Ningxia Hui and YRI indicated the maximum differentiation (0.10129), and the average F_ST_ value between Chinese Ningxia Hui and CHB indicated the least differentiation (0.00108).

**Table 3:** Genetic Differentiation between Subpopulations.

	CEU	TSI	YRI	ASW	JPT	CHB	Chinese Ningxia Han	Chinese Ningxia Hui
CEU	0.00000	0.00592	0.16061	0.11947	0.11528	0.11038	0.10627	0.09615
TSI		0.00000	0.15190	0.11445	0.10791	0.09906	0.09690	0.09125
YRI			0.00000	0.00440	0.09563	0.09941	0.08699	0.10129
ASW				0.00000	0.07961	0.08586	0.07391	0.08105
JPT					0.00000	0.00970	0.00920	0.01131
CHB						0.00000	0.00000	0.00108
Chinese Ningxia Han							0.00000	0.00363
Chinese Ningxia Hui								0.00000

Abbreviations are as follows: CEU, Utah residents with Northern and Western European ancestry; TSI, Toscani in Italy;YRI, Yoruba in Ibadan; ASW, African Ancestry in Southwest US; JPT, Japanese in Tokyo; CHB, Han Chinese in Beijing.

F_ST_ values were also calculated for each SNP to quantify the differences among the CHB, Hui, and Han populations (Table 4). Eighteen SNPs showed low F_ST_ values (F_ST_≤0.004) between the CHB and Chinese Ningxia Han samples. By contrast, the F_ST_ values of five SNPs between the Hui and Han populations ranged from 0.000 to 0.050, suggesting the existence of genetic differentiation between the two populations. The other thirteen SNPs had F_ST_ values ≤0.000 between the two populations (Table 4).

**Table 4:** Fst and Allele frequency of 18 SNPs from cancer-related genes in CHB, Chinese Ningxia Han, Chinese Ningxia Hui.

rsID	Gene	Alleles	f_CHB	f_Chinese Ningxia Han	Fst	p value	χ2	p value	f_Chinese Ningxia Han	f_Chinese Ningxia Hui	Fst	p value	*χ2*	*p value*
rs1042522	TP53	C>G	0.451	0.445	-0.004	0.910	0.021	0.884	0.445	0.414	-0.002	0.496	0.451	0.502
rs2395655	CDKN1A	G>A	0.485	0.448	-0.001	0.396	0.669	0.413	0.448	0.424	-0.003	0.541	0.276	0.599
rs3176320	CDKN1A	A>G	0.228	0.248	-0.003	0.739	0.267	0.605	0.248	0.278	-0.002	0.613	0.532	0.466
rs3829963	CDKN1A	C>A	0.432	0.397	-0.002	0.505	0.626	0.429	0.397	0.399	-0.004	0.991	0.003	0.957
rs3829964	CDKN1A	C>T	0.296	0.317	-0.003	0.631	0.252	0.616	0.317	0.313	-0.004	0.991	0.009	0.924
rs4135234	CDKN1A	G>A	0.117	0.117	-0.004	0.991	0.001	0.980	0.117	0.101	-0.003	0.694	0.315	0.575
rs762624	CDKN1A	C>A	0.388	0.403	-0.004	0.784	0.115	0.735	0.403	0.384	-0.003	0.694	0.189	0.664
rs730506	CDKN1A	G>C	0.107	0.131	-0.001	0.550	0.665	0.415	0.131	0.172	0.002	0.198	1.548	0.213
rs4488809	TP63	T>C	0.490	0.466	-0.003	0.586	0.296	0.586	0.466	0.439	-0.003	0.604	0.324	0.569
rs13042395	C20orf54	C>T	0.272	0.217	0.004	0.117	1.969	0.161	0.217	0.207	-0.004	0.820	0.073	0.788
rs13361707	PRKAA1	C>T	0.505	0.545	-0.001	0.369	0.772	0.380	0.545	0.414	0.029	0.000	8.040	0.005
rs17728461	LIF downstream 38kb	C>G	0.228	0.252	-0.003	0.604	0.365	0.546	0.252	0.222	-0.002	0.468	0.562	0.454
rs2274223	PLCE1	A>G	0.189	0.207	-0.003	0.622	0.233	0.629	0.207	0.131	0.015	0.045	4.630	0.031
rs465498	CLPTM1L	A>G	0.165	0.190	-0.002	0.577	0.495	0.482	0.190	0.121	0.013	0.054	4.062	0.044
rs753955	MIPEP-TNFRSF19	A>G	0.350	0.303	0.001	0.297	1.170	0.279	0.303	0.439	0.035	0.000	9.454	0.002
rs9485372	TAB2	G>A	0.432	0.417	-0.004	0.775	0.108	0.742	0.417	0.449	-0.002	0.468	0.499	0.480
rs9841504	ZBTB20	C>G	0.107	0.131	-0.001	0.496	0.665	0.415	0.131	0.101	0.000	0.324	1.013	0.314
rs9934948	LOC100506172	C>T	0.519	0.541	-0.003	0.595	0.233	0.629	0.541	0.495	0.000	0.360	1.017	0.313

Abbreviations are as follows: CHB, Han Chinese in Beijing.

The allele distribution information of eighteen SNPs among the eight populations was statistically significant (p<0.05), as shown in Table 5. For example, the p53 codon 72 (rs1042522) G allele showed a distribution of 24.2% in CEU, 27.6% in TSI, 63.9% in YRI, 59.8% in ASW, 31.7% in JPT, 45.1% in CHB, 41.4% in Chinese Ningxia Hui, and 44.5% in Chinese Ningxia Han.

**Table 5:** Allele frequency of 18 SNPs from cancer-related genes in study.

rsID	Gene	Alleles	f_CEU	f_TSI	f_YRI	f_ASW	f_JPT	f_CHB	f_Chinese Ningxia Han	f_Chinese Ningxia Hui	*χ2*	*p value*
rs1042522	TP53	C>G	0.242	0.276	0.639	0.598	0.317	0.451	0.445	0.414	113.125	0.000
rs2395655	CDKN1A	G>A	0.586	0.593	0.194	0.246	0.346	0.485	0.448	0.424	117.872	0.000
rs3176320	CDKN1A	A>G	0.389	0.336	0.495	0.582	0.389	0.228	0.248	0.278	82.716	0.000
rs3829963	CDKN1A	C>A	0.076	0.220	0.417	0.311	0.385	0.432	0.397	0.399	98.903	0.000
rs3829964	CDKN1A	C>T	0.535	0.435	0.093	0.139	0.212	0.296	0.317	0.313	138.358	0.000
rs4135234	CDKN1A	G>A	0.071	0.131	0.056	0.082	0.188	0.117	0.117	0.101	24.843	0.001
rs762624	CDKN1A	C>A	0.702	0.752	0.431	0.451	0.462	0.388	0.403	0.384	124.997	0.000
rs730506	CDKN1A	G>C	0.263	0.154	0.273	0.303	0.125	0.107	0.131	0.172	51.383	0.000
rs4488809	TP63	T>C	0.485	0.472	0.343	0.270	0.543	0.490	0.466	0.439	36.377	0.000
rs13042395	C20orf54	C>T	0.051	0.061	0.009	0.016	0.284	0.272	0.217	0.207	148.727	0.000
rs13361707	PRKAA1	C>T	0.268	0.290	0.361	0.328	0.548	0.505	0.545	0.414	79.840	0.000
rs17728461	LIF downstream 38kb	C>G	0.258	0.313	0.060	0.156	0.192	0.228	0.252	0.222	50.880	0.000
rs2274223	PLCE1	A>G	0.313	0.393	0.356	0.369	0.207	0.189	0.207	0.131	69.307	0.000
rs465498	CLPTM1L	A>G	0.429	0.439	0.606	0.549	0.130	0.165	0.190	0.121	254.324	0.000
rs753955	MIPEP-TNFRSF19	A>G	0.657	0.645	0.083	0.230	0.370	0.350	0.303	0.439	229.810	0.000
rs9485372	TAB2	G>A	0.187	0.140	0.269	0.230	0.457	0.432	0.417	0.449	109.866	0.000
rs9841504	ZBTB20	C>G	0.126	0.056	0.315	0.279	0.216	0.107	0.131	0.101	86.454	0.000
rs9934948	LOC100506172	C>T	0.141	0.145	0.421	0.451	0.558	0.519	0.541	0.495	177.218	0.000

Abbreviations are as follows: CEU, Utah residents with Northern and Western European ancestry; TSI, Toscani in Italy; YRI, Yoruba in Ibadan; ASW, African Ancestry in Southwest US; JPT, Japanese in Tokyo; CHB, Han Chinese in Beijing.

The allele frequency and relative physical coordinates of the eighteen SNPs are shown in Table 4. The allele frequencies of all eighteen SNPs were found to be highly similar between the Chinese Ningxia Han and CHB samples, showing no significant difference between the two populations (p>0.05). However, four SNPs showed significantly different genetic distributions between Chinese Ningxia Hui and Chinese Ningxia Han (p<0.05). For example, the frequencies of the rs13361707 T, rs2274223 G, and rs465498 G alleles in Chinese Ningxia Hui (0.414, 0.131, and 0.121, respectively) were significantly less than those in Chinese Ningxia Han (0.545, 0.207, and 0.190, respectively). The frequency of the rs753955 G allele in Chinese Ningxia Hui (0.439) was significantly greater than that in Chinese Ningxia Han (0.303), and the frequencies of the other fourteen SNPs showed no significant differences between Chinese Ningxia Hui and Chinese Ningxia Han (p>0.05).

## Discussion

Molecular genetics studies in the last few decades have provided the basis for ancestral analysis and analysis of the geographic origins of human populations using genetic data. Starting approximately 100,000 years ago, anatomically modern humans migrated out of East Africa and gradually spread to South Asia, Australia, Europe, East Asia, and eventually the Americas. All people living today are direct descendants of these earlier humans. Populations living in different parts of the world today exhibit a small number of genetic differences due to migration, mutation, genetic drift, natural selection, and reproductive isolation [25].

In this study, we sought to investigate the diversity pattern of cancer-related genes between Hui and Han Chinese from the Ningxia region of China to explore the hereditary differences between the two populations. We selected eighteen SNPs from cancer-related genes for the analysis. We first used F_ST_ to measure the degree of population differentiation [26], and our results suggested that all SNPs of the cancer-related genes differ substantially among the eight populations. In addition, all F_ST_ results were consistent with the allele frequency comparisons among the different populations.

We then used average F_ST_ values to investigate the diversity pattern of cancer-related genes among eight subpopulations with respect to the eighteen SNP sites. We found that there was little genetic differentiation between any two European, African, or East Asian groups, although there was remarkable genetic differentiation between each pair of the aforementioned ethnic groups.

We subsequently investigated the genetic differentiation between CHB and Han Chinese in Ningxia. Our data indicated that the allele frequencies of all eighteen SNPs were very similar between the two populations. The Han Chinese population is generally thought to be naturally divided by the Yangtze River into two groups: the Southern Han and Northern Han groups. A previous study showed that the difference between these two groups of Han Chinese is greater than that between a given subpopulation and ethnic minorities at the same location [27]. Because CHB and Ningxia Han Chinese are both located in northern China, the genetic difference between CHB and Ningxia Han Chinese should be smaller than that between the Southern Han and Northern Han groups.

Our study further showed that the allele frequencies of four SNPs differentiated Ningxia Hui Chinese from Ningxia Han Chinese, indicating that there was some genetic differentiation in the distribution of four SNPs from cancer-related genes between Chinese Ningxia Hui and Han. Among four SNPS, SNP rs753955 had been reported to be associated with lung cancer in Chinese Han population by GWAS. SNP rs753955 was also found to be related to non-cardia gastric cancer in Chinese Ningxia Han in our previous study [19]. However, their associations with cancer in Ningxia Hui people are still unclear. The Chinese Hui ethnic group descended from Arab and Persian Muslim immigrants who came to China and married local girls hundreds or even thousands of years ago [28]. However, Hui people adhere to Islamic principles [29]. To retain religious purity and group identity, most Hui people have always isolated themselves socially from other people in enclaves. Hui marriage practices tend toward endogamy in all respects, especially in the rural part of Ningxia. Therefore, the Hui population is religiously and culturally conservative [30]. Consequently, our results show that the allele frequency distribution of four SNPs in some cancer-related genes in the Ningxia Hui Chinese is different from that in Ningxia Han Chinese, indicating that hereditary differences exist between Hui and Han Chinese in Ningxia. Shuhua Xu et al. systemically investigated the influence of admixture on the diversity of absorption, distribution, metabolism, and excretion (ADME) genes responsible for drug absorption, distribution, metabolism and excretion in five northwestern Chinese minority populations, namely Tajik, Uyghur, Kazakh, Kirgiz and Hui. They found that northwestern Chinese populations exhibited substantial differences in some ADME genes compared with Han Chinese [31]. Therefore, both the work of Xu and our research indicate that Hui Chinese are different from the Han Chinese population with respect to their genetic background. Previous studies have shown that genetic background diversity might result in differences in disease spectrum. For example, in two large studies from Korea and China, Pro/Pro at p53 codon 72 (rs1042522) was found to be associated with colon cancer; the respective frequencies of the Pro allele in cases and controls were 34.0% and 36.4% in Koreans and 50.3% and 39.6% in Chinese [32]. In two larger studies, one with 442 cases and 904 controls in the United States, the frequency of the Pro allele in cases and controls was 27.4% and 25.5%, respectively [2]. Another study with 352 cases and 316 controls in Spain showed Pro allele frequencies in cases and controls of 24.0% and 21.0% and found no association between the p53 codon 72 polymorphism and the risk of colorectal cancer [33]. These results highlight that ethnicity is a critical factor in the distribution of allele frequencies, which may ultimately affect a person’s cancer spectrum. These results will have significant implications when evaluating cancer susceptibility, sensitivity to radiotherapy and chemotherapy, and prognosis in Hui and Han Chinese.

## Conclusions

Our results showed for the first time that Hui Chinese in Ningxia exhibit differences in certain cancer-related genes compared with Han Chinese in Ningxia. Therefore, we suggest that population differences in cancer susceptibility, the efficacy of cancer therapy, and prognosis should be carefully considered. However, there are some limitations to our study, such as the small sample size, the small number of genetic markers, and the very limited geographic location. In addition, although we have investigated the associations between some of the SNPs and cancer in Ningxia Han population [19], we could not further compare the associations between the genetic polymorphisms and cancer among the Hui and Han populations due to a lack of cancer samples from Hui people, which would greatly strengthen this work. Therefore, additional research is needed to further identify genetic differences between the Hui and Han populations.

## Supporting Information

S1 Table The primers for polymerase chain reaction (PCR) and probers for LDR. (DOC)
