# Supplementary material for: Comparison of Genetic Variants in Cancer-Related Genes between Chinese Hui and Han Populations
Source: PLoS One. 2015 Dec 18;10(12):e0145170. doi: 10.1371/journal.pone.0145170 (PMC4684198; doi:10.1371/journal.pone.0145170)
Supplement: S1 Table — (DOC) [file pone.0145170.s001.doc]

S1 Table. The primers for polymerase chain reaction (PCR) and probers for LDR.

| rsID | Primers or probes | Sequences |
| --- | --- | --- |
| rs1042522 | Upstream primer | AGAAGATGACAGGGGCCAGGAG |
|  | Downstream primer | TCCCAAGCAATGGATGATTTGA |
|  | FG | TCTCTCGGGTCAATTCGTCCTT TGCTGGTGCAGGGGCCAAGG |
|  | FC | TGTTCGTGGGCCGGATTAGT TGCTGGTGCAGGGGCCAAGC |
|  | FP | GGGGAGCAGCCTCTGGCATT TTTTTTTTTT |
| rs2395655 | Upstream primer | GGGAGGTCAGGGGTGTGAGGTA |
|  | Downstream primer | GGGAAGGAGGGAATTGGAGAGA |
|  | RG | TTCCGCGTTCGGACTGATAT CACCCTACACTCACCTGAACAGAAGACAC |
|  | RA | TACGGTTATTCGGGCTCCTGT CACCCTACACTCACCTGAACAGAAGACAT |
|  | RP | CCCTGYGGTTGCAGCAGCTT TTTTTTTTTTTTT |
| rs3176320 | Upstream primer | GTGTGCTGCGTTCACAGGTGTT |
|  | Downstream primer | TGGTGGACACAGTGGCGTAAAG |
|  | FG | TCTCTCGGGTCAATTCGTCCTT TAAGTGCGCGGGTGACGTGG |
|  | FA | TGTTCGTGGGCCGGATTAGT TAAGTGCGCGGGTGACGTGA |
|  | FP | GTCGGGATGTGCCGGAGAYC TTTTTTT |
| rs3829963 | Upstream primer | CAGGAGACCTCTAAAGACCCCAGGT |
|  | Downstream primer | CCCAGACACACTCTAAGGGAGGA |
|  | RC | TTCCGCGTTCGGACTGATAT TCGCAAGGATCTGCTGGAAG |
|  | RA | TACGGTTATTCGGGCTCCTGT TCGCAAGGATCTGCTGGGAT |
|  | RP | ATCACATACCCTGTTCAGAGTAACAGGC TTTTTTTTTTT |

| rs3829964 | Upstream primer | CAGGAGACCTCTAAAGACCCCAGGT |
| --- | --- | --- |
|  | Downstream primer | CCCAGACACACTCTAAGGGAGGA |
|  | FC | TTCCGCGTTCGGACTGATAT CAGCCKGCTCCCTTGCCATC |
|  | FT | TACGGTTATTCGGGCTCCTGT CAGCCKGCTCCCTTGCCGTT |
|  | FP | TTCAGGGCAGARGTCCTCCC TTTTTTT |
| rs4135234 | Upstream primer | CTTGGGAGCCTGTGTGAAGGTG |
|  | Downstream primer | GGACAAAATAGCCACCAGCCTCT |
|  | RG | TTCCGCGTTCGGACTGATAT CAACATGTTGGGACATGTTCCTCAC |
|  | RA | TACGGTTATTCGGGCTCCTGT CAACATGTTGGGACATGTTCCTCAT |
|  | RP | RGCCAGAAAGCCAATCAGAGC TTTTTTTTTT |
| rs762624 | Upstream primer | GGGAGGTCAGGGGTGTGAGGTA |
|  | Downstream primer | GGGAAGGAGGGAATTGGAGAGA |
|  | RG | TCTCTCGGGTCAATTCGTCCTT CCCGTATACACTGCTGGGGAATCG |
|  | RA | TGTTCGTGGGCCGGATTAGT CCCGTATACACTGCTGGGGAACCT |
|  | RP | GGGGCTCAGAGAAGTCTGGTGTTTTTTTT |
| rs730506 | Upstream primer | GGGGTGTCTAGGTGCTCCAGGT |
|  | Downstream primer | GGACACATTTCCCCACGAAGTG |
|  | FG | TCTCTCGGGTCAATTCGTCCTT GGTGCTTCTGGGAGAGGTGACCAAG |
|  | FC | TGTTCGTGGGCCGGATTAGT GGTGCTTCTGGGAGAGGTGACCAAC |
|  | FP | TGAGGGATCAGTGGGAATASAGG TTTTTTTTTTT |

| rs9485372 | Upstream primer | ACAGGGCCGCTCTCAGTCAGT |  |
| --- | --- | --- | --- |
|  | Downstream primer | TGCCCCATGCATAGGTGCTTAG | |
|  | RG | TTCCGCGTTCGGACTGATAT TCTCTCCACAGGGAATAGTGATATGTTGC | |
|  | RA | TACGGTTATTCGGGCTCCTGT TCTCTCCACAGGGAATAGTGATATGTCGT | |
|  | RP | AGTTTAAGAATGCACAATGYCTTTATTTTTAACA TTTTTTTT | |
| rs9934948 | Upstream primer | TGCAATGAACCAAAGGCAAATG | |
|  | Downstream primer | TGGCCTGTTTCCCTCTATCTGTG | |
|  | FC | TTCCGCGTTCGGACTGATAT GGGATTTTGGAGTTGTTTATATAGCATGTCACC | |
|  | FT | TACGGTTATTCGGGCTCCTGT GGGATTTTGGAGTTGTTTATATAGCATGTCACT | |
|  | FP | TTATCTGTRTAAAGCAAAGAACATCTCCAGG TTTTTTTTTT | |
